# Redefining Diabetes Care: Evaluating the Impact of a Carbohydrate-Reduction, Health Coach Approach Model in New Zealand

**DOI:** 10.1155/jdr/4843889

**Published:** 2024-12-24

**Authors:** Caryn Zinn, Jessica L. Campbell, Marina Po, Losi Sa'ulilo, Lily Fraser, Glen Davies, Marcus Hawkins, Olivia Currie, David Unwin, Catherine Crofts, Nigel Harris, Tom Stewart, Grant Schofield

**Affiliations:** ^1^Human Potential Centre, Faculty of Health and Environmental Sciences, Auckland University of Technology, Auckland, New Zealand; ^2^Turuki Healthcare, Mangere, Auckland, New Zealand; ^3^Reversal NZ, Taupo, New Zealand; ^4^Highbrook Medical Centre, Highbrook, Auckland, New Zealand; ^5^Real Healthy Me, Christchurch Central, New Zealand; ^6^Norwood Surgery, Southport, UK

**Keywords:** carbohydrate reduction, health coaching, health system transformation, lifestyle medicine, prediabetes, primary care, sustainability in healthcare, T2D management

## Abstract

This study explores a novel healthcare model employed in the primary care setting integrating a carbohydrate-reduction dietary approach and health coaching for managing prediabetes (PD) and Type 2 diabetes (T2D) in New Zealand. Using qualitative methods, we conducted focus groups with 46 patients and individual interviews with health coaches and general practitioners across two regions. Five major themes emerged from inductive thematic analysis: reduced carbohydrate lifestyles, health coaching, implementation, empowerment, and sustainability. Patients reported significant health improvements, including weight loss, reduced medication burden, and increased energy. Challenges included resistance from some medical professionals and negative public perceptions. Health coaching played a crucial role in patient care, providing individualised support and enhancing health literacy. The study found that this model both improved patient outcomes and also alleviated the burden on healthcare professionals by managing time-intensive aspects of patient care. Barriers to the adoption of this model include scepticism about low-carbohydrate diets and the need for more education and awareness among healthcare professionals. The findings suggest that this healthcare model has the potential to transform the management of PD and T2D in primary care, shifting patients from lifelong medication dependence to significant health improvements and potential disease remission or reversal.

## 1. Introduction

Type 2 diabetes (T2D) represents a substantial and growing global health challenge [1], straining healthcare systems and frequently leading to a range of complications [2, 3]. While traditionally prediabetes (PD) and T2D have been regarded as progressive, chronic ailments necessitating lifelong management, emerging research indicates that these conditions can be reversed or put into remission through dietary and lifestyle changes. Although there are several dietary approaches that can be used to manage PD and T2D [4], reducing overall carbohydrate intake shows the best evidence for improving glycemia [5–9], even independently of weight loss [10]. Although reduced carbohydrate diets have been incorporated into national diabetes dietary guidelines worldwide for several years [11–13], this has not yet formally taken place in New Zealand (NZ) [14]. However, the uptake and practice of this approach are rapidly expanding in primary care in NZ, as demonstrated by our ongoing work in this area [15]. This paper addresses the preventable and potentially reversible nature of PD and T2D by examining a primary care strategy focused on whole food and carbohydrate reduction for diabetes management and reversal. The model is supported by holistic healthcare delivery, based on the health coach approach.

The significance of addressing PD and T2D lies not only in their high prevalence but also in the significant socioeconomic burdens they impose. Managing chronic diseases places a substantial burden on healthcare systems and individuals [3, 16]. In NZ, as in many other parts of the world, the escalating diabetes epidemic calls for innovative and sustainable solutions, particularly in light of concerning figures around general practitioner (GP) burnout, shortages, and intention to retire [17]. Moving away from a system that is heavily reliant on GPs and instead utilising other allied healthcare professionals may, therefore, have multiple advantages, particularly given the current constraints faced by the usual 15-min GP appointments. Health coaching is a relatively new concept in NZ and, until recently, has not been widely embedded in the primary healthcare system. However, substantial development in growing this workforce has meant an increasing number of GPs are now able to refer patients to health coaches and wellbeing advisors, called Health Improvement Practitioners. These healthcare providers are now employed either within a GP clinic or in a Primary Health Organisation (a cluster of clinics which work together to care for patients who are registered with them) [18, 19]. While the importance of educating individuals to manage their diabetes has been recognised since the 1930s [20], current healthcare models often fall short in equipping patients with the skills and motivation essential for effective and sustained diabetes management through lifestyle interventions [3, 20]. While tight metabolic control can delay or prevent diabetes complications, the motivation and ability of patients to take on this responsibility vary greatly [21]. Consequently, significant segments of the diabetic population remain underserved, and the nature of healthcare delivery is critical if it is to be successful in bringing about sustained positive health outcomes. Given that health coaching is based on the principles of behaviour change and emphasizes a personalised approach with regular patient interaction, it presents a promising solution for managing PD and T2D through dietary and lifestyle changes. By customizing intervention strategies to meet the specific needs and circumstances of each patient, health coaching is aimed at enhancing patient engagement and, importantly, improving adherence, thereby eliciting sustained outcomes.

This study explores the experiences of patients with PD and T2D, along with healthcare professionals, in a holistic care model centred on whole-food carbohydrate reduction. The model is holistic and multidisciplinary and combines a health coaching approach with a carbohydrate-reduction dietary approach. In this context, health coaching represents a style of working with individuals to facilitate positive changes in their health and well-being. It encompasses a personalised and culturally sensitive approach, acknowledging the complexity of behavioural change and embedding it in their practice while respecting individual differences. Health coaching necessitates collaborative efforts between patients and healthcare providers, empowering patients with the knowledge to take control of their own lives. Working within this comprehensive approach, our study is aimed at better understanding the effectiveness of the model in empowering patients, healthcare practitioners, and the healthcare system to manage diabetes effectively. Further, it is aimed at gaining insights into the feasibility of scaling up this model.

## 2. Materials and Methods

### 2.1. Model Characteristics in Two NZ Practices

The study examined two distinct healthcare systems in NZ—one public and one private. While each system operates uniquely, they share several common elements in their approach to managing T2D. Key facts are displayed in Box 1 with full details.

#### 2.1.1. Practice 1

This primary care private practice is spearheaded by a GP with over 30 years of experience in medicine. In the past 3 years, they integrated a therapeutic carbohydrate-reduction eating and coaching approach into their practice and regularly consult with patients via GP appointments. Additionally, the GP conducts weekly educational meetings, focusing on diabetes management and the benefits of a reduced carbohydrate diet for reversing the condition. Complementing these efforts, patients have the opportunity to work closely with a health coach upon GP referral, further individualizing their care and enhancing their understanding and application of these dietary principles. The patient population is predominantly NZ European with a range of sociodemographic backgrounds.

#### 2.1.2. Practice 2

This publicly funded primary care practice is a Māori Provider Trust based on indigenous principles offering family-based wellness and social services. The diabetes care model is led by a GP and Clinical Director who has been a proponent of carbohydrate reduction as a dietary approach for diabetes management since 2015. Once a GP has seen the patient, they refer them to the health coach and accompanying supporting initiatives. These services are comprehensive and offer patients access to health coaches, biweekly support groups conducted by the clinical director, and weekly cooking classes focused on reduced carbohydrate recipes. The practice also offers a broad spectrum of programmes, encompassing various aspects of well-being, not limited to dietary interventions. All of these services are available to patients as part of their enrolment at the practice. This multifaceted approach not only caters to the medical needs of patients but also addresses the cultural and community aspects of health, ensuring a holistic and culturally sensitive treatment pathway. Patients at this practice predominantly identify as belonging to Māori and Pacific Islander communities, with the practice located in an area characterised by lower socioeconomic status.

### 2.2. Data Collection

The study employed a mixture of one-on-one interviews with healthcare professionals and focus groups with patients. The one-on-one interviews were conducted virtually, while focus groups were held in person, in each respective region of NZ. All focus groups and interviews were carried out between November 2022 and April 2023. Participation was based on voluntary decisions, and informed consent was provided before all scheduled interviews and focus group sessions. Ethical approval for this study was granted by the Auckland University of Technology Ethics Committee (AUTEC), reference number 22/253.

### 2.3. Focus Groups

The focus groups were designed with a semi-structured approach, enabling comprehensive discussions on the experiences of patients with the model of care and their perspectives on the reduced carbohydrate approach. Focus groups included patients from both practices. In all cases, participants were greeted by members of the research team who identified with both Māori and Pacific Island ethnicities. They then welcomed the participants in their native languages using traditional protocols to open the focus group and allow cultural connection. As a token of appreciation of the participants' time, a small gift of petrol vouchers and *What the Fat!*—a published book on carbohydrate reduction authored by the lead researchers of this study—were provided. From Practice 1, 22 patients participated and were divided into five focus groups (two groups of six patients, two groups of three, and one group of four). From Practice 2, 24 patients participated and were divided into four focus groups (comprising 10, six, four, and four patients, respectively). Patients were recruited via advertisements placed on social media and messaging platforms. The recruitment for focus groups continued until data saturation was achieved, which occurred after the completion of these sessions.

### 2.4. Interviews With Health Professionals

Parallel to patient focus groups, one-on-one interviews were conducted with health professionals from each practice. Interviews were held with two professionals (one doctor and one health coach) from Practice 1 and four professionals (one doctor and three health coaches) from Practice 2.

### 2.5. Data Analysis

All interviews and focus groups were recorded and transcribed verbatim using Otter AI Pro software Version 3.44.2-240223-4aa344c2, followed by manual verification by a member of the research team. The transcripts were then analysed using inductive thematic analysis via NVivo analytic software (Release 1.6.1 (1137), QRS International Pty Ltd). Data analysis was independently carried out by two authors (L.S. and M.P.). To ensure the reliability and validity of the thematic analysis, two additional authors (C.Z. and J.L.C.) reviewed all identified themes, refining categories through merging and subdividing where necessary.

Quotations used throughout this report have been lightly edited to facilitate readability and to maintain the anonymity of participants/individuals mentioned by participants.

## 3. Results

Focus group participants spanned a wide range of ages and ethnic backgrounds; demographic characteristics are detailed in Table 1.

**Figure 1:**
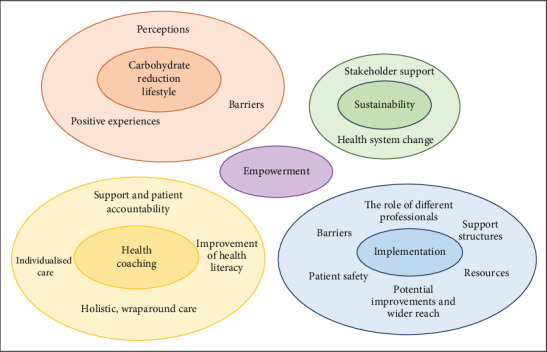
Key themes and subthemes from patients and healthcare practitioners.

The inductive analysis identified five major themes: (1) reduced carbohydrate lifestyles, (2) health coaching, (3) implementation of the model, (4) empowerment, and (4) sustainability of the model. Within these broad categories, additional subthemes were identified, as presented in Figure 1.

Key Themes and Discussion Points

Throughout each section of the results, each theme is described from the perspective of both patients and health practitioners; accompanying quotes are presented in Tables 2, 3, 4, 5, and 6.

### 3.1. Theme 1: Reduced Carbohydrate Lifestyle (Diet and Lifestyle Approach)

#### 3.1.1. Perceptions of Reduced Carbohydrate Diets

Patients transitioning to reduced carbohydrate diets experienced a significant shift in their dietary habits and gained a new understanding of what constitutes healthy food for T2D, challenging conventional beliefs about low-fat diets being the healthier dietary option. This was also noted in relation to the carnivore (meat only) diet, which several patients were following despite them initially having reservations about eating so much meat.

#### 3.1.2. Positive Experiences

Patients implementing a reduced carbohydrate diet reported numerous benefits, including significant weight loss without hunger, a healthier, addiction-free relationship with food, and more stable energy levels for both themselves and their families. Many individuals observed substantial improvements in HbA1c levels and were able to reduce or discontinue medication. The diet also had a positive impact on a range of other health conditions, including heart palpitations and polycystic ovarian syndrome.

All health coaches emphasised their role in creating a positive patient experience by helping patients find enjoyable foods and make small changes within culturally accepted dietary habits. Healthcare professionals also spoke about positive changes they had observed in patients, including improvements in glycaemic control, body weight, and the extent to which medications were needed. They further believed that the approach should be more widely utilised, and that the health system needed to recognise carbohydrate reduction as an effective method for treating and managing PD and T2D.

#### 3.1.3. Barriers

Patients identified multiple barriers to adopting or maintaining a reduced carbohydrate eating approach. Key issues included scepticism, resistance, and lack of up-to-date knowledge from healthcare professionals (particularly GPs and dietitians), and national organisations such as the New Zealand Society for the Study of Diabetes (NZSSD), Diabetes New Zealand, and the National Heart Foundation of New Zealand. Additionally, patients noted a lack of consensus and varying opinions within the low-carbohydrate community, causing confusion about optimal dietary choices. The influence and conflicts of interest from the food and pharmaceutical industries were a significant concern, alongside the cost of lower carbohydrate products. Patients suggested that more support from supermarkets, restaurants, and cafes was needed.

Social challenges included negative perceptions from friends and family, with concerns about health impacts and disapproval of dietary choices. Special occasions such as holidays and social gatherings posed particular difficulties, with the temptation of high carbohydrate foods and social pressure to conform.

Healthcare professionals also highlighted the reluctance of some GPs to endorse carbohydrate-reduction approaches. The divergence from traditional dietary guidelines was seen as the main reason for this. Health professionals agreed that the carbohydrate-centric focus of both the national dietary guidelines and the diabetes-specific dietary guidelines in NZ were one of the biggest barriers faced, alongside the noted lack of support from GPs and dietitians.  Table 2 presents supporting quotes aligning with these subthemes.

### 3.2. Theme 2: Health Coaching

#### 3.2.1. Individualised Care That Is Culturally Appropriate

Patients with PD and T2D highly valued individualised care, where health coaches tailored their approach to each individual within a general framework. They appreciated the one-on-one attention, problem-solving, attention to detail, and thorough explanations. Patients also noted the genuine interest displayed by health coaches and their consideration of the individual's cultural background.

Healthcare professionals stressed that personalising care was essential for success and very different from standard interactions between GPs and patients. Individualising care was felt to increase the accessibility of behavioural change, for example, by creating budget-friendly meals and helping patients to access local food while considering transportation limitations. Similarly, health coaches strongly stressed the importance of cultural appropriateness in healthcare. They further noted that being a health coach, who was reflective of the community they serve, was seen as an advantage. More generally, healthcare professionals highlighted the adaptability of their approach to suit the backgrounds of their patients.

#### 3.2.2. Holistic, Wraparound Care

Patients appreciated the comprehensive approach of health coaches, which included not only dietary guidance but also cultural practice and other lifestyle factors. Some noted utilising other programs on offer via the primary healthcare provider which were not related to diet or diabetes but were nevertheless beneficial. Patients also valued that the approach extended to treating other members of their family/household.

Healthcare professionals also stressed the need for a holistic care model that prioritizes individual well-being, emphasizing the importance of addressing the comprehensive needs of not just patients but also their families. This approach includes flexibility, adaptability, and the provision of free care in some instances. Additionally, health coaches highlighted the introduction of various health behaviours to enhance well-being beyond diabetes management, with attention to cultural considerations.

#### 3.2.3. Support and Patient Accountability

Patients highlighted the significance of accountability and the role of support from both groups and health professionals in maintaining it. They valued the encouragement to take personal responsibility and the opportunity to share progress. The proactive engagement and consistent support from health coaches, along with the ease of communicating updates, feelings, and dietary choices via email, were particularly appreciated.

Healthcare professionals acknowledged that behavioural change, particularly related to nutrition and lifestyle, is a significant challenge. They recognised the importance of a supportive environment and stressed the role of peers in making the process easier. They also found that maintaining patient commitment was crucial for success, and regular checkups helped keep patients motivated and accountable for their goals. Nevertheless, the patient's own commitment and willingness to embrace change were noted as key determinants of their success in improving their health.

#### 3.2.4. Improvement of Health Literacy

Gaining a greater understanding of their medical condition and nutrition was deemed critical by patients, and many appreciated exploring the science behind diabetes. Regular meetings were seen as particularly beneficial, offering engaging and stimulating discussions with repetitive reinforcement of key information. Several patients discussed how the information they were given acted as a springboard to pursuing their own research, with educational resources like informative videos playing a crucial role in enhancing their knowledge and self-accountability.

Healthcare professionals described how patients often leave conventional medical appointments without a full understanding of their medical condition. They emphasised the importance of health coaches in bridging the gap in health literacy and providing patients with the knowledge required to manage their conditions effectively. This was especially important given the low health literacy in many communities, even though multiple family members may be suffering from diabetes.  Table 3 presents supporting quotes aligning with these subthemes.

### 3.3. Theme 3: Implementation

#### 3.3.1. Barriers

Patients expressed various obstacles to accessing care, for example, several were unaware of all the services and support available, including the existence of a Facebook group and weekly meetings. The biggest barrier was the financial aspect of the model, with GP appointments being prohibitively costly in the private system.

Healthcare professionals spoke about barriers in relation to the contradiction between the NZ nutrition guidelines and the carbohydrate-reduction approach. This caused tension among clinicians. Additionally, health coaches noted that some patients found greater comfort in dealing with traditional medical professionals over health coaches, partly due to confusion about the distinct roles of health and life coaches. Funding concerns were also prominent, with apprehensions about political influences and inconsistent funding, especially given the proximity to an upcoming election. The need for stable resources for health coach employment was stressed, along with uncertainties surrounding health coaching pay scales compared to other allied health professionals.

#### 3.3.2. Resources

Patients appreciated the available resources and support but had specific suggestions to improve the implementation of the healthcare model in other locations. Some desired resources that were tailored to their specific needs and circumstances, highlighting the need for practical tools that could be personalised. Patients envisioned a central repository of resources accommodating diverse lifestyles, including working individuals, those with limited time, families, and various cultural backgrounds. They suggested the creation of a problem-solving forum, professionally produced resources for common issues, and practical aids like posters, tiered guides for sugar alternatives, and cheat sheets for making healthier choices at different eateries.

Healthcare professionals echoed the desire for more resources, including materials and visual representations to enhance patient understanding. Providers acknowledged that patients often seek validation and verification, for example, requesting meal plans despite there being a wide range of recipes and meal plans available on the Internet. The potential benefits of continuous glucose monitors (CGMs) were mentioned, with one health coach noting that three-monthly HbA1c testing was too out of reach for some patients as they required more regular feedback on progress. Lack of funding for CGMs was the current constraint to these being made widely available.

#### 3.3.3. Support Structures

Patients identified key support structures essential for their engagement in healthcare, including dedicated GPs, group meetings that fostered self-discipline and community, group chats, and health coach check-ins. The importance of family involvement was emphasised, and for those without supportive families, a buddy system was suggested.

GPs recognised the significance of health coaches in managing lifestyle medicine clinics. Similar to patients, they also highlighted the role of community support systems, particularly through group meetings and digital platforms.

#### 3.3.4. Patient Safety

Patient safety was not frequently spoken about by the patients themselves, but one participant noted their negative experience of stopping all medication abruptly.

In contrast, patient safety is a paramount concern to healthcare professionals within the context of implementing a new model of healthcare. GPs particularly emphasised the significance of safety around medication when transitioning patients to a reduced carbohydrate diet, noting that safe deprescribing was essential yet was an often inadequately taught skill.

As mentioned previously, some healthcare professionals acknowledged the apprehension among other practitioners regarding the safety of therapeutic carbohydrate reduction, particularly when concerns arise about perceived potential risks like heart attacks.

#### 3.3.5. Potential Improvements and Wider Reach

In addition to the previously mentioned resources, patients proposed improved communication about the full range of services and support available when starting the program. Some patients expressed a desire for the inclusion of exercise programs and classes within the program to enhance accountability, although this varied by location, with certain health coaches with fitness backgrounds already incorporating such elements.

Healthcare professionals discussed various strategies for expanding the reach of the model of care to a broader audience including exploring opportunities within Māori and Pacific communities and initiatives like community gardens. Additionally, they noted that accommodating different schedules by providing more flexible hours would be beneficial moving forward. Some individuals also noted that they would like to introduce exercise elements to health coaching, concurring with patient feedback.

#### 3.3.6. The Role of Different Professionals Within the Model

The involvement of various professionals in the healthcare model was more frequently addressed by healthcare professionals than by patients. Patients' views on the importance of different professionals varied regionally, with some emphasizing health coaches and others relying more on GPs, particularly where GPs led weekly group sessions.

A collaborative approach between GPs and health coaches was emphasised. GPs were seen as crucial at the start of a patient's journey, particularly for safe prescribing and deprescribing before transitioning to a reduced carbohydrate approach with health coach guidance. The potential integration of health psychologists and dietitians was also discussed, with the former able to address the psychological aspects of patients' health journeys, while dietitians could assist complex patients with specific dietary needs. However, there was tension noted between health professionals, with dietitians sometimes resisting reduced carbohydrate approaches and concerns about health coaches overlapping with dietitians' traditional roles.  Table 4 presents supporting quotes aligning with these subthemes.

### 3.4. Theme 4: Empowerment

Patients reported feeling empowered through realizing that they could make meaningful changes in their own lives. For many, having choices was crucial, especially when they have previously been told they had no option but to take medication in managing their PD or T2D.

Health professionals also spoke about patient choice being key and about the significance of patients going on a journey with a group of others who were working towards shared goals. They also reported having undergone a transformation in their own approach to the practice of medicine. One GP noted a shift from conventional practice to addressing the underlying causes of health issues.  Table 5 presents supporting quotes aligning with these subthemes.

### 3.5. Theme 5: Sustainability

#### 3.5.1. Health System Change

Patients recognised the challenges that could impede the success and sustainability of this approach, particularly in relation to the role of conventional doctors. Most of their concerns were linked with resistance to carbohydrate-reduction approaches and preventative medicine more broadly, as already discussed.

Healthcare professionals also recognised the need for greater understanding of the importance and impact of lifestyle and preventive medicine. GPs discussed how the model could address issues such as short GP appointments, GP burnout, and the shortage of GPs. By delegating detailed nutritional and lifestyle guidance to allied health professionals and health coaches, GPs could reduce their workload and manage their time more effectively within the typical 15-min consultation model. Health coaches were considered instrumental to the model's long-term success, and it was suggested that having more health coaches than doctors in the longer term could be desirable.

Sustainability was also linked with the need for comprehensive data collection on patient outcomes to help secure future funding for health coaching. Additionally, health coaches discussed strategies to improve their efficiency, such as grouping patients based on specific needs like sleep quality and exercise habits, allowing them to maximize their impact with limited time.

#### 3.5.2. Stakeholder Support

Patients emphasised the necessity for enhanced support from the food industry and educational systems for any sustained success of lifestyle medicine to occur. They praised local efforts, like cafes offering reduced carbohydrate options, and advocated for the widespread availability of healthier food choices, especially in areas with lower socioeconomic status. The discussion also touched on the potential for supermarkets to play a significant role in making reduced-carb and low-sugar alternatives more accessible.

Healthcare providers also identified the challenges arising from external factors, primarily related to the national and diabetes nutrition guidelines which have a significant impact on clinical practices and public health policies. As noted earlier, there were also some concerns about funding support being withdrawn from health coaching programs when the government changed.  Table 6 presents supporting quotes aligning with these subthemes.

## 4. Discussion

The purpose of this study was to explore the experiences of patients with PD or T2D, as well as health professionals involved in a holistic model of care based on whole-food carbohydrate reduction. While there were some variations in views between patients and healthcare professionals, there was a consensus on the success of the model and the reasons why it was felt to work. Key findings included significant health improvements reported by patients, including weight loss, better glycaemic control, and increased energy levels. Health coaching emerged as a critical component, facilitating regular, personalised interactions that contributed to patient empowerment and autonomy. Barriers included resistance from some medical professionals and public perceptions about carbohydrate reduction, as well as financial barriers affecting access to healthy food options and GP appointments. Despite these barriers, this holistic model shows promise for managing and potentially reversing PD and T2D, advocating for a shift in healthcare practice towards lifestyle medicine delivered via a health coaching approach.

### 4.1. Reduced Carbohydrate Approaches

Despite the overwhelmingly positive experiences and outcomes of participants using a therapeutic carbohydrate-reduction approach both in this study and more broadly [5–9, 22], the approach is still met with widespread scepticism and resistance from health professionals in NZ [14], including GPs and dietitians. Accordingly, there is a critical need for more widespread education and awareness around the growing literature on the efficacy of therapeutic carbohydrate reduction for diabetes management [4–7, 9, 23, 24]. The ostensible resistance is evidently owing to concerns about the high saturated fat content of some reduced carbohydrate diets, the potential impact on LDL cholesterol, and, consequentially, cardiovascular health. Recent debates have challenged the traditional view that saturated fat increases the risk of cardiovascular disease [25–28]. While uncertainty remains, there is a need for nuanced distinctions between different types of saturated fats [29] and their diverse food sources, with a greater focus on overall dietary patterns [28–30]. It is further important to interpret lipid markers in the context of overall health rather than in isolation. Evidence suggests that within the context of a reduced carbohydrate diet replete in fibre, vitamins, and minerals [31] and low in ultraprocessed foods, blood lipid markers may in fact undergo positive changes [6] including over long time frames [32], particularly in individuals who are overweight or obese [33]. This should be an area of focus in helping patients with PD and T2D.

Another point of resistance asserted was the lack of formal endorsement by NZSSD. While international consensus guidelines in many countries/regions including the United States, Canada, Europe, the United Kingdom, and Australia now endorse carbohydrate reduction as a legitimate option for the management of T2D [11–13], there remains considerable work to do in NZ to bring about a similar shift in perspective [14]. It is likely that some resistance to this dietary approach will persist until concerns about lipid profiles and official guidelines are addressed or resolved. For patients in the present study, however, in the face of such challenges, good adherence to this eating approach was maintained. This was facilitated by their experience of improved health outcomes, alongside strong support structures, including health coaches, peer groups, and family involvement, which have been recognised for their effectiveness in diabetes management [16].

When comparing our results to global trends in diabetes care, carbohydrate-reduction models have been integrated into healthcare systems with varying levels of success. In the United States, the Virta Health initiative has shown significant long-term improvements in glycaemic control and medication reduction through a continuous remote care model [23, 24, 34]. The use of frequent digital monitoring tools, including CGMs, allows for real-time feedback and higher patient adherence. However, Virta Health's status as a private entity allows for greater accessibility to these advanced technologies. In contrast, within NZ's government-subsidised healthcare system, limited access to CGMs and other monitoring technologies may hinder the immediacy of patient feedback and overall success. Nevertheless, regular check-ins with health coaches can go some way to bridging this gap.

Similarly, in the United Kingdom, Dr David Unwin's approach within the National Health Service (NHS) has demonstrated success in helping patients achieve drug-free remission of T2D through a low-carbohydrate dietary intervention [6, 7]. Unwin's model emphasizes patient education and the use of visual aids to illustrate carbohydrate's effects on blood glucose, which has improved patient outcomes significantly. Similar to our findings, regular check-ins and phone calls to motivate patients if they start to deviate from their dietary goals are instrumental and can rapidly get patients back on track [6].

Despite these successes, resistance to or refusal to recommend reduced carbohydrate diets remains relatively widespread [22, 35]. Previous studies examining patient experiences of managing T2D with reduced carbohydrate diets have also found that they frequently report scepticism resistance and a lack of knowledge from their GPs and other health professionals [22]. Much like in the present study, patients report that they previously experienced being automatically placed on medication, rather than being given the guidance needed to reverse their diabetes via diet and lifestyle. Despite this, both patient and practitioner experiences of reduced carbohydrate diets in the context of diabetes management have been extremely positive [22, 35]. As in the current study, practitioners have previously reported that they were finally able to change patients' lives after years of failure to do so when only recommending the conventional higher carbohydrate, low-fat diets [35].

### 4.2. Health Coaching and Sustainability of the Model

Both patients and health professionals were universally positive about the health coaching approach studied in the two models of care. The effectiveness of health coaching in improving outcomes for patients with various chronic diseases has previously been documented [36–39]. With emphasis on personalised, regular interactions that foster accountability, reduce anxiety, and promote sustained self-care, health coaching can bring about behaviour change in a way that standard models of care rarely achieve [40]. A shift from a passive acceptance of pharmacological interventions to actively making lifestyle choices is facilitated when healthcare providers support patient autonomy, leading to increased patient self-confidence [41]. This highlights the importance of a patient-centred approach in healthcare [42]. In this regard, giving patients sufficient knowledge to be able to make informed choices is critical, which is difficult in standard 15-min GP appointments [43] and likely explains the rarity of sustained behaviour change in the conventional model. Integrating more health coaches into the existing model of care could manage these time-intensive aspects of patient care, significantly lessening the burden on GPs while still providing this personalised support. This need for more nonclinical staff such as health coaches has been highlighted in other recent work in an NZ context, with clinical staff expressing that a greater number of nonclinicians could ease the T2D workload burden [43, 44]. This shift could move patients from being life-long dependents on medication to individuals who are able to reverse chronic conditions and regain their health. Similarly, this transition redefines the role of GPs from being prescribers of medication to facilitators of holistic health and well-being, reducing workload in the longer term. Given the figures around GP burnout and retirement [17], this is significant. The approach, therefore, has the potential to optimize healthcare delivery in a system currently under significant stress, while mitigating the economic burden on the health system by decreasing long-term healthcare costs and medication use.

Although there are challenges in scaling this model nationwide, particularly in terms of health coach funding and integration, progress has already been made. Notably, since the focus groups were conducted, funding for health coaches in NZ has improved, and, as seen in our engagement with multiple primary care settings across NZ, they are becoming better integrated into the healthcare system. This positive trend is encouraging, as it reflects growing recognition of the role of health coaches in supporting sustained behaviour change and improving patient outcomes, allowing more patients to benefit from this approach. It is essential, however, that training sufficiently equips health coaches with the behaviour change skills and relevant nutrition knowledge required to be effective. This is unfortunately still inconsistent in NZ and is an area for development. Collection of data and dissemination of results related to the success of health coaching approaches may further be helpful in securing more funding moving forward. In the long term, financial implications for the healthcare system are likely to be positive. Preventing the progression from PD to T2D, as well as reducing disease-related complications among those already diagnosed with T2D, could lead to significant cost savings. Although no formal financial modelling has been done, it is reasonable to assume that fewer patients with advanced T2D would alleviate some of the burdens on the healthcare system. This has been demonstrated at Norwood Surgery in the United Kingdom, where Dr Unwin's practice reported significant savings of public health funds due to a reduction in diabetes medications through the use of a low-carbohydrate dietary approach [7]. Furthermore, if this approach proves more accessible or palatable to underserved populations such as Māori and Pasifika, it could have broader implications for improving health outcomes for these groups.

### 4.3. Future Improvements in the Implementation of the Model

In addition to the health system changes noted above involving reorientation of the workforce to include more health coaches, updated guidelines, and greater acceptance of reduced carbohydrate approaches, our study highlights other areas for improvement in order for the model to be implemented at broad scales.

Firstly, creating resources to address the varied needs of patients managing chronic conditions is crucial. In an era saturated with information on the internet, establishing reliability becomes challenging, and patients may frequently place greater value on centrally provided resources and information [45]. In response to the feedback from focus groups, we have developed extensive resources to support patients and health professionals, including a dedicated website offering recipes, practical tips for reducing carbohydrates on a budget, guidance for those with limited time, culturally tailored advice, and relevant scientific literature. While the website is associated with our implementation science research, it is free and accessible to anyone wishing to learn more about reduced carbohydrate eating patterns [46].

Second, there is a great need for culturally safe and inclusive healthcare solutions that reach underserved communities who face health disparity [43, 44, 47, 48]. In an NZ context, this includes engagement with Māori and Pacific communities to identify champions within their own communities to lead lifestyle changes. In agreement with previous research [43], we found that engagement and understanding improved when health coaches or other health professionals came from similar ethnic backgrounds. The model's alignment with Te Whare Tapa Whā, a holistic Māori model of health, further supports its cultural relevance [49, 50]. This health framework encompasses four dimensions: taha tinana (physical health), taha hinengaro (mental health), taha wairua (spiritual health), and taha whānau (family health), and allows for a culturally resonant approach that addresses the values and needs of Māori populations.

More broadly, tailoring health coaching practices to different cultural contexts would enhance the practical applicability of this model across diverse patient populations. For example, health coaching approaches could be adapted to reflect the cultural values, dietary traditions, and communication styles of various ethnic and immigrant groups, ensuring that advice is relevant and accessible. In cultures where family and community play a central role in decision-making, health coaches could engage family members in lifestyle changes to improve adherence and outcomes, as was evident in the present study. Additionally, offering health education materials and resources in multiple languages and using culturally appropriate metaphors or examples would further support inclusivity. Developing cultural competency training for health coaches would ensure that they are equipped to address the unique needs of different populations, making the model adaptable and effective across a wide range of healthcare settings.

Finally, there is an evident need for a shift in the role of dietitians. Collaborative efforts between dietitians and health coaches could bridge gaps in health literacy and cultural sensitivity, with each profession bringing different strengths. This partnership could enhance chronic disease management by ensuring consistent, evidence-based dietary guidance. However, the resistance within NZ's dietetic profession towards adopting carbohydrate-reduction approaches, despite evidence, suggests a need for a paradigm shift in both training and guidelines to fully embrace this model in PD and T2D management.

### 4.4. Overcoming Barriers

In addition to the challenges discussed above, this study identified other significant barriers to the adoption of a reduced carbohydrate model and health coaching approach. Financial constraints were frequently noted by patients, including the high cost of lower carbohydrate food options and the expense of GP appointments. While the website we developed offers practical tips for reducing carbohydrates on a budget, larger scale policy interventions such as subsidies for healthier food and more widespread affordable healthcare services are still needed to facilitate broader adoption of the model. These challenges are not specific to reduced carbohydrate approaches, and patients following alternative diabetes diets have also noted difficulties in accessing healthy food options [51].

In accordance with previous research [22], social factors also posed challenges for patients, as several reported feeling social pressure from family and friends who were sceptical of the health impacts of carbohydrate reduction. As with patients on a variety of other diabetes diets [51], special occasions, such as holidays and gatherings, presented specific difficulties, with patients feeling obligated to consume high-carbohydrate “treat” foods in order to conform. To help patients navigate these challenges, structured support strategies could be implemented, including role-playing social scenarios in health coaching sessions to help patients practice declining food or explaining their dietary choices. We have also developed specific resources, such as a “cheat sheet” that provides practical guidance on the most and least favourable reduced-carbohydrate options at various takeaway outlets across NZ including bakeries, Chinese, Italian, and fast-food restaurants—which can empower patients to make informed choices even in social or convenience-driven situations.

Finally, the holistic nature of this healthcare model, which aligns with the principles of Te Whare Tapa Whā and incorporates family and community in the lifestyle change process, may in itself reduce scepticism and help overcome barriers to its adoption, particularly as positive health outcomes are achieved.

### 4.5. Study Strengths and Weaknesses

One of the primary strengths of this study lies in its comprehensive exploration of a novel model of care for diabetes management, particularly highlighting the integration of health coaching and a reduced carbohydrate eating approach. This research adds significant value to the field by focusing on patients with both diagnosed PD and T2D, the former being a group that is often underrepresented in diabetes research and has traditionally been underserviced within the healthcare system [52]. The inclusion of diverse perspectives from both patients and healthcare professionals (doctors and health coaches) enriches the study's findings, offering a well-rounded understanding of the model's impact. Further, patients and practitioners from a range of cultural and ethnic backgrounds were included, with patients covering a wide range of ages. While the patient sample was small relative to the entire patient population that has experienced this model of care, we achieved saturation in data collection from the focus groups, ensuring comprehensive coverage of information.

The study is not without limitations. The self-selecting nature of the participants who volunteered for the focus groups has the potential to introduce a selection bias. Participants who are more positively inclined towards the model of care, depending on their success, might have been more motivated to participate, potentially skewing the data. However, it was noted that not all patients had experienced success stories with their diabetes outcomes, indicating that patients were able to separate their experiences of the model of care from their treatment outcome. This was encouraging as it indicated a more authentic critique of the model independent of the influence of outcomes. It also allowed for insights into how the model of care adapted to situations of patient failures. Moreover, even when previous studies have tried to recruit practitioners with negative experiences of delivering reduced carbohydrate guidance, they have found overwhelmingly positive experiences, suggesting this may not reflect a biased sample but rather be representative of widespread experiences [35]. Nevertheless, patients included in the study were those who made a choice to manage their PD or T2D via diet and lifestyle measures. As such, they likely represent a subset of patients who are highly motivated to make changes. This reflects the real-world scenario, where individuals who opt for lifestyle interventions are typically those already inclined towards making such changes. Consequently, the focus on motivated patients aligns with the population to whom this model would realistically apply. While randomised controlled trials have demonstrated the efficacy of reduced carbohydrate approaches under controlled conditions, our study examines the real-world effectiveness of this model. As such, the absence of less motivated individuals does not detract from the relevance of these findings for the target group that is open to lifestyle-based management.

### 4.6. Research Implications and Future Research

The findings of this study have significant implications for the healthcare system in NZ and elsewhere, particularly in the context of the challenges faced by high numbers of GPs nearing retirement [17]. Diabetes poses a growing global challenge, with over 500 million individuals affected as of 2021, and projections indicate that this will rise to over 1.3 billion by 2050 [1]. This highlights the urgent need for innovative approaches to address the escalating burden of diabetes, particularly given the lack of progress in diabetes reversal in mainstream healthcare delivery. A system and health professional role reorientation towards a more holistic model, incorporating established community support structures and grounded in behaviour change principles, may be timely.

In response to the growing need for evidence on the long-term impact of this approach, future research will focus on expanding the scope of this model across more primary care clinics in NZ. We are currently undertaking a wider study that will examine the transition to reduced carbohydrate approaches combined with health coaching in a larger and more diverse set of clinical settings [46]. This work will involve collecting comprehensive data on patient outcomes, including glycaemic control, weight management, and medication reduction, to evaluate the long-term sustainability and scalability of the model. These data will also provide insights into the success factors and barriers within real-world settings, further informing the integration of this model into the healthcare system.

## 5. Conclusions

In summary, this study identifies both the promises and challenges associated with the healthcare model under investigation. Adopting a carbohydrate-reduction approach delivered significant health benefits for patients but was met with barriers, including resistance from healthcare professionals and social perceptions. Health coaching proved to be an invaluable component of the model, offering individualised care and support based on the framework of behaviour change and cultural responsiveness, while also addressing the need for increased health literacy. To ensure sustainable implementation, the model requires enhanced education for healthcare professionals, comprehensive data on patient outcomes, and increased public awareness. Importantly, this study demonstrates the potential for a paradigm shift from a pharmaceutical-based system to one prioritising lifestyle medicine, empowering patients to regain control over their health and reducing the burden on primary care systems managing lifestyle-related conditions like T2D. Future research should explore scaling this model and evaluating its long-term impacts.

## Figures and Tables

**Box 1:**
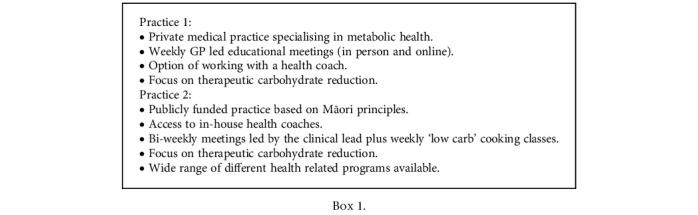


**Table 1:** Demographic details of focus group participants from each practice.

**Feature**	**Practice 1 (** **n** = 22**)**	**Practice 2 (** **n** = 24**)**
Female gender; *n* (%)	14 (61%)	17 (71%)
Age (years); mean (SE)	67 (±1.7)	50 (±2.4)
Age range (years)	49–79	34–64
Ethnicities	NZ European (12), other European (6), South African (2), Australian/European (1), and Māori/NZ European (1)	Māori (7), Māori/NZ (7), Pacific Islands (6), Pacific Island/European (1), Pacific Island/Māori (2), and Pacific Island/Indian (1)

**Table 2:** Subtheme quotes from Theme 1: Reduced carbohydrate lifestyle (diet and lifestyle approach).

**Subtheme**	**Patient quotes**	**Health practitioner quotes**
Perceptions of reduced carbohydrate diets	“We're finding the [Low-carb High fat] food really yummy, it's nice. Our treat...and it's the same one all the time and I'd never get sick of it, and that's the chocolate avocado mousse…. I love it now.”	
	“The programme's really helped me, especially with food because I did not know what food was good or bad.”	
	“… Break a lot of those stories or those conditionings of you know, to be healthy, you have to eat a lot of fruit, lots and lots of high carb stuff that we did not realize is just becoming part of our addiction.”	

Positive experiences	“Two weeks after trying that diet. Boom, 7 kilo drop. 7 kilos. I never got that kind of drop.”	“We have a huge Polynesian, Māori whānau [family] sort of demographic in this area, I am able to talk about kai [food] that that really relates to them and then how they can switch that meal to still be low carbohydrate by taking a few ingredients out [and] replacing certain ingredients with other options, so that they are still maintaining the flavours.”
	“I've been doing it for a year and a half, and slowly my diabetes has come from 110 [mmol/mol HbA1c] and I'm now a year and a half sitting at 36 [mmol/mol].”	“Diabetes and how it's another pandemic on its own and how effective LCHF [low carbohydrate, healthy fat] is, in treating that and managing it. Our health system needs reform in recognising that.”
		“There's just this dramatic change. And he's like ‘oh I've just been doing what you said with the diet'.”

Barriers	“When I went to my GP [location removed], he was surprised at how far my sugar levels had dropped. And he said, ‘what were you doing?' And I said, ‘I'm doing the low-carb, following low-carb,' and he was a bit sceptical.”	“I think in the end it comes down to the cardiovascular implications, eating more saturated fat, and potentially raising the LDL, I think that's the big stumbling block, I think they are scared that they might cause someone to have a heart attack.”
	“I've experienced the fact that my GPs did not know what they were talking about when it came to low [carb]. They did not have the latest information. They were not up on anything.”	
	“Pretty much you can Google any opinion of what you want to hear and find that. You know, and it's like, so you'll, you know, flip through, like maybe 3 or 4 different keto sites find one that says yes you can have ice cream and think 'I knew it.”	
	“I just get really annoyed that the big food companies and the pharmaceutical companies are just screwing everything, they want you sick, they want you sick, so they can make profit and that's all it's about, it's money.”	

**Table 3:** Subtheme quotes from Theme 2: Health coaching.

**Subtheme**	**Patient quotes**	**Health practitioner quotes**
Individualised care that is culturally appropriate	“Talking with him and [name removed] actually, it was inspiring, actually, and I felt quite validated.”	“The actual foundation of it does not need to change; the approach that we use, it's just we have to adapt it to suit the environment they come from.”
	“His one-on-one attention to you when you are speaking to him. You know, he's, he's genuinely interested, and will try and problem solve with you.”	“…. A lot of the things that I used to say [to him]…..'you think of yourself as a Toa. So, Toa is like a chief'. And I'm like, ‘you are chief, the chief cannot run the family if he's not 100% inside', so I'm talking about his health. And then he was like, ‘okay, so Coke, Sprite, Mountain Dew, I should not be drinking that'. I'm like, ‘no……You're already sweet yourself. You do not need to add more sugar…'”
		“I think, being a Māori health provider that is delivering to the Māori and Pacific Island community... I feel more connected to the community itself…… I think that that's really important, you know, that the work we are representative or reflective of is our community that we serve.”

Holistic, wraparound care	“And then he'd send me some maramataka stuff [Māori lunar calendar] as well, to help me understand well what's going on, in the maramataka that could impact on how I'm feeling…. connected to the moon and the tides……. it's really good, especially with energy.”	“The model that we are using now is kind of this wraparound care model where we just really care for them personally. So, we have got follow-ups built in.”
	“I really love what she said. She says, ‘So tell me your story.' And I thought, oh my gosh, that's really cool…. it's a holistic approach… they asked about your trauma and your stress. And it was just really cool how she explained it.”	“Our health coaches are really flexible in the way that they work with whānau [family]. So, you know, they will they certainly meet the person wherever they want to meet, they try their hardest to work with the whole household if possible.”

Support and patient accountability	“Kept going to the meetings, which I absolutely think, for me, I'm one of those people. I need support…. Yeah, accountability, that like, I had to be responsible. How would I be responsible if I did not go to it?”	“Nutrition change and lifestyle change is hard, but the more people around you that do it, and respect it, and understand it, the easier it becomes.”
	“He's [health coach] proactive... they really do make an effort. They do not just say ‘well, this is what you need to do, see you later'. They are just like, ‘we are gonna keep on top of you'.”	“When I do checkups on them, it's like, ‘Have you cheated this week? Like, you know, have you had any carbs?' And they are like, ‘Yeah, I fell off the waggon. But then I jumped back on, my BSL [Blood sugar levels] was this' and I'm like, ‘Yeah, well, what's your goal? What was your goal when you came in a month ago?'”

Improvement of health literacy	“The biggest thing that's helped me is learning about carbs. And the simple fact that they turn to sugar in the body.”	“The doctors only get a certain amount of time with the patients. And sometimes the patients go away, not really knowing what their medical condition is.”
	“They're really worth going to you know, even though we have the same pattern of what he's [GP] going to talk about, we still learn something quite totally different each time even though it's a similar topic.”	“For a lot of people as well, there is a very low health literacy. So, helping them to understand, you know, what their condition is, is another big part of some of the conversations that we have.”
	“Little mini videos [on YouTube] which have been really, really helpful... it just keeps me more conscious about staying on track.”	

**Table 4:** Subtheme quotes from Theme 3: Implementation.

**Subtheme**	**Patient quotes**	**Health practitioner quotes**
Barriers	“I'd love to be going to [GP] but I cannot afford it. I do not have the funds to cover that.”	“So sometimes the ‘oh you are a health coach, what does that mean?' Oh, and people still get it mixed up with life coach as well, which is quite interesting.”
	“When you asked about the hospital food, it's like, they do not even have a diabetic diet up there.”	“The biggest barrier is the ministry of health nutrition guidelines. I just think, until that's explicitly supportive of low carb, you are going to have tension from the clinicians.”

Resources	“I'm running a business, I'm 60 years old, I do not have the time to, and I sort of need resources available to me, for people in my situation.”	“I wish we had more materials and more resources…. I think just having some of those materials would be handy or resources like having a visual representation of how much sugar is in this bottle of coke.”
	“It'd be really cool to have a toolkit of things that people can choose what they need.”	
	“I think it'd be great if you had like, people who work and they have their forum because they [are] all having similar problems.”	

Support structures	“It's about the awhi [help] too you know, like [we] can support each other. And knowing that like you are not on that journey alone.”	“Because of the reality of people actually committing to doing it, I found that when we did not have health coaches, I got very little result.”
		“It's such a huge thing, type two [diabetes], that the more support … the more groups of people that you can get together, I think the better.”
Patient safety	“I went cold turkey with medication, which was really dangerous because I took a lot of medication out, that caused major problems.”	“Obviously, we do not want our patients to be harmed.... we have to be kind of hyper vigilant on the safety netting.… I think the health coaches, working with any patients on meds need a clinical supervisor, they need someone that can make sure that those things are happening, because that's not their job to understand the medications.”
		“The thing that I'm talking to our GPs about the most is actually just safe prescribing…. [deprescribing] is a really important skill that's not taught that well.”

Potential improvements and wider reach		“You could introduce, you know, community gardens to a Marae [Māori sacred place] setting. And then, you know, preparing the kai (food).”
		“We should be going to see different iwi [Māori tribes], and say, how we want to start training champions in each iwi, like health coach champions.”
		“We look at our Polynesian whānau [family]…. you know, going to some of the churches, the big church groups and working with them, that can have some massive [impact], because they'll see the benefits. And I think there's a big opportunity there…. When you change one church group, you'll change another big one.”

**Table 5:** Subtheme quotes from Theme 4: Empowerment.

**Patients**	**Health practitioners**
“For me, it was just the realization that I could actually effect change myself; a realization I had thought unattainable before.”	“I come from a place of giving people options, and, letting them make their own reality call at that moment around what's going to work with them… I'm always saying, there's two approaches here and you we can do mixtures of both... like the food, you are going to be able to potentially reduce medication, or come off medication or lose weight, or come off blood pressure meds, or I can give you another tablet…. what's gonna work for you today?”
“I was like, okay, well, now I need to kind of start eliminating certain foods…… because I do want to lose weight as well, because I'm 60 [years of age] and I kinda need a plan to get old.”	“It's changed my practice, I've gone from being a typical ineffective doctor, prescribing medications for the symptoms to now a moderately effective doctor, dealing with the cause of the problem.”

**Table 6:** Subtheme quotes from Theme 5: Sustainability.

**Subtheme**	**Patients**	**Health practitioners**
Health system change	“Because you can do this personally. But if you go to an ordinary GP, you are going to be prescribed metformin.”	“So, I'm always talking about nutrition, with patients...since we have had health coaches, I do not go into the ‘nitty gritty' so much as what I'm used to.”
	“Until we have got doctors on-board. People are getting mixed messages.”	“There would be a solution to the doctor to burn out…this model of having doctors supported by health coaches in a lifestyle medicine clinic, in every town is a solution to the healthcare crisis. And the biggest solution is that it actually cures people.”
		“The idea is similar to the Vaers reporting system for vaccine injury. Everyone who has reversed their diabetes and come off the medication… we can report that to an online database and the clients can do it. …and we start then observing that there are thousands of people that have reversed their chronic condition. …. I'd like to know the method, if it was with a vegan diet, or if it was with exercise or, with low-carb or keto.”
		“Since we got our first in-house health coach in 2020, that's where I've seen the change, person by person, people actually getting results in a way that GPs cannot.”
Stakeholder support	“You've got so many alternatives in the States [USA] for food choices when it comes to getting foods that comply…. but you do not have the same choices here.”	“The biggest barrier is the Ministry of Health nutrition guidelines. I just think, until that's explicitly supportive of low carb, you are going to have tension from the clinicians.”
	“The biggest thing anyone can do is to change the supermarkets, change the food that is all wrapped up.”	

## Data Availability

Data are contained within the article.
